# Statistical theory of branching morphogenesis

**DOI:** 10.1111/dgd.12570

**Published:** 2018-10-24

**Authors:** Edouard Hannezo, Benjamin D. Simons

**Affiliations:** ^1^ IST Austria Klosterneuburg Austria; ^2^ The Wellcome Trust/Cancer Research UK Gurdon Institute University of Cambridge Cambridge UK; ^3^ Wellcome Trust Centre for Stem Cell Research University of Cambridge Cambridge UK; ^4^ Cavendish Laboratory Department of Physics University of Cambridge Cambridge UK

**Keywords:** biophysical concepts, mammary gland, morphogenesis, statistical model, stem cell

## Abstract

Branching morphogenesis remains a subject of abiding interest. Although much is known about the gene regulatory programs and signaling pathways that operate at the cellular scale, it has remained unclear how the macroscopic features of branched organs, including their size, network topology and spatial patterning, are encoded. Lately, it has been proposed that, these features can be explained quantitatively in several organs within a single unifying framework. Based on large‐scale organ reconstructions and cell lineage tracing, it has been argued that morphogenesis follows from the collective dynamics of sublineage‐restricted self‐renewing progenitor cells, localized at ductal tips, that act cooperatively to drive a serial process of ductal elongation and stochastic tip bifurcation. By correlating differentiation or cell cycle exit with proximity to maturing ducts, this dynamic results in the specification of a complex network of defined density and statistical organization. These results suggest that, for several mammalian tissues, branched epithelial structures develop as a self‐organized process, reliant upon a strikingly simple, but generic, set of local rules, without recourse to a rigid and deterministic sequence of genetically programmed events. Here, we review the basis of these findings and discuss their implications.

## 1 INTRODUCTION

To sustain life, organisms must exchange nutrients and metabolic waste products with the environment. In unicellular organisms, such as bacteria or yeast, where the surface to volume ratio is high, the surface of the cell is large enough to meet these demands. However, in multicellular organisms, such as mammals, strategies must be developed to maximize the area of surfaces where such exchange can occur. In the small intestine, this challenge is met by organizing the epithelium into an array of finger‐like protrusions, known as villi, which extend into the gut lumen. In volumnar tissues, such as kidney, lung, mammary gland, prostate and pancreas, exchange surfaces are packed efficiently around ramified branched epithelial networks. How do these structures form? How do instructions encoded at the molecular and cellular scale translate into the large‐scale organization of complex branched epithelia? This is the problem of branching morphogenesis (Iber & Menshykau, 2013) and is exemplified by the pubertal development of the mouse mammary gland epithelium (Sternlicht, 2005).

**Figure 1:**
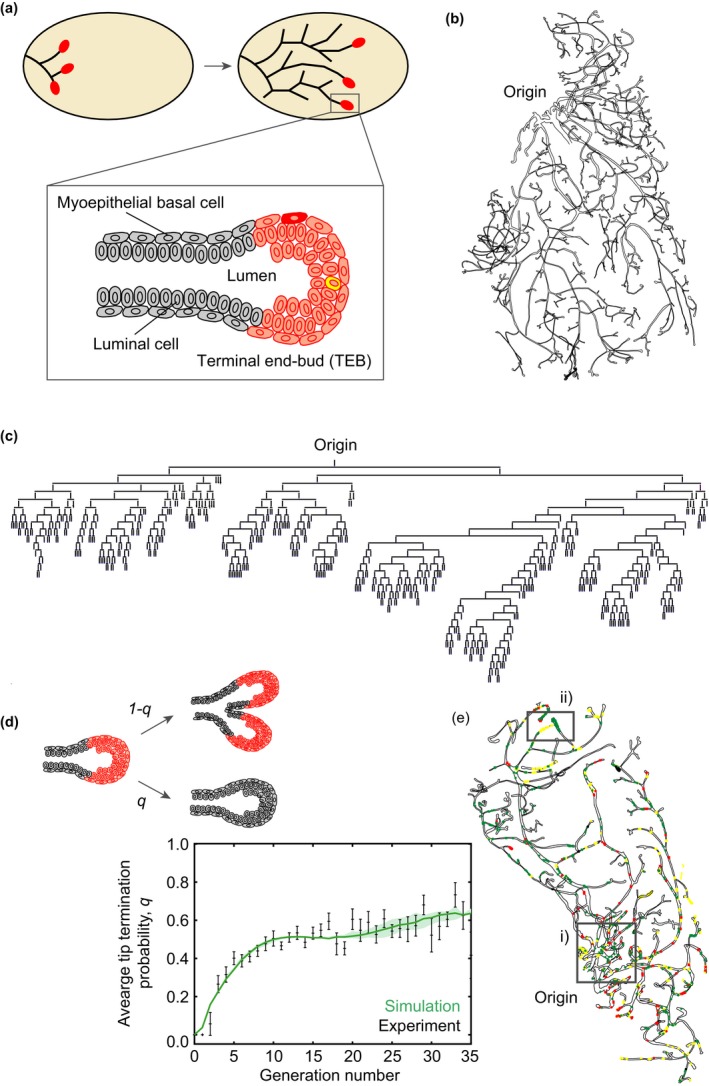
Embryonic development of mouse mammary gland epithelium. (a) At birth, the mouse mammary gland epithelium forms a rudimentary ductal tree‐like structure (upper‐left panel). Expansion of the ductal epithelium is driven by proliferative cells at the ductal tips (marked in red), that drive a sequential process of ductal elongation and bifurcation (upper‐right panel). As epithelial cells exit the ductal tip – known as the terminal end‐bud – they exit cell cycle giving rise to a simple bilayer epithelium comprised of luminal cells and myoepithelial basal cells (bottom panel). (b) Outline of the ductal epithelial network of a mouse at the end of puberty (8 weeks), when it has expanded to fill a fat pad. (c) Topology of the corresponding ductal network showing that some subtrees terminate early while others go through multiple rounds of division. (d) Schematic (upper panel) shows that ductal subtrees can be segmented as a sequence of collective fate decisions in which active terminal end‐buds choose stochastically between termination (cell cycle exit), with probability *q*, and bifurcation, with probability *1‐q*. Averaging over multiple terminal end‐buds, the probability *q* is shown empirically to converge towards *q = 1/2* (points). The line shows the result of a numerical simulation of the model discussed in the main text and Figure 2a. (e) Map of labelled epithelial cells marked using a multicolor mouse confetti reporter system induced at 3 weeks and fixed at 8 weeks. Box (i) shows a matrix of quiescent cells labelled in the pre‐existing network at the induction time. Box (ii) shows the clonal outputs of labelled mammary stem cells illustrating how repeated bouts of ductal bifurcation leads to an enrichment of individual clones marked by a single confetti color. Panels (b,c,e) are adapted from Figures presented in Scheele et al., 2017; while panel (d) is reproduced from Hannezo et al., 2017

In mouse, the mammary glands are specified along the ventral epidermis around embryonic day (E)12 as placode‐like structures that sprout and invade an adipocyte‐rich stroma. At birth, the mammary gland comprises a small rudimentary tree‐like structure involving a minimally branched network (Figure 1a). Then, during puberty, cellular precursors – termed “mammary stem cells” – drive a serial process of ductal bifurcation and elongation, leading to the specification of a complex ramified ductal network that extends to fill the fat pad (Figure 1a). In adult, hormonal changes through the estrous cycle promote bouts of alveoli growth and regression while, in pregnancy, alveoli mature into the milk‐producing glands. The ducts form a simple stratified epithelium comprised of an outer layer of myoepithelial basal cells and an inner layer of luminal cells (Figure 1a). During puberty, measurements based on short‐term incorporation of thymidine analogues shows that the ductal growth is driven by actively cycling progenitors positioned at or near ductal tips – known as “terminal end‐buds” – localizing the mammary stem cell population to these sites.

What is the molecular identity, sublineage potential, and fate behavior of mammary stem cells during pubertal development? Are they stem cells at all? How do stem cells and their progeny integrate fate choice with collective cell rearrangements to direct the large‐scale patterning of the ductal network? And are these mechanisms conserved in the patterning of other branched epithelia?

Traditionally, to address the mechanisms that regulate mammary gland development, most studies focus on the repertoire of transcription factors and signaling pathways that regulate cell fate behavior in the terminal end‐buds (Macias & Hinck, 2012). But, to address factors that regulate the spatio‐temporal patterning and large‐scale organization of tissue, these may not be the most useful starting variables. Instead, to resolve the factors that control collective cell fate behavior and patterning, it makes sense to start by considering the larger‐scale structural organization of the complex ductal network. Recently, by combining lineage tracing strategies with morphometric measurements of the ductal network structure, recent studies have provided evidence of a conserved mechanism of branching morphogenesis in the mouse mammary gland (Hannezo et al., 2017; Scheele et al., 2017), kidney (Hannezo et al., 2017) and pancreas (Sznurkowska et al., 2018). Here, we review the basis of these findings and discuss their wider implications.

## 2 THE LARGE‐SCALE ORGANIZATION OF THE MAMMARY GLAND DUCTAL NETWORK IS PREDICTED BY A SIMPLE STATISTICAL RULE

First, to define quantitatively the large‐scale structure of the mouse mammary gland epithelium, the ductal organization was traced from whole‐gland reconstructions of tissue acquired at the end of puberty and stained for the ductal basal cell marker Keratin 14 (Figure 1b). The results underline a remarkably complex arrangement, with ductal networks adopting a non‐stereotypic organization (Lu, Sternlicht, & Werb, 2006), foliating into a diversity of subtrees of variable size and topology: After several rounds of near‐symmetrical dichotomous branching, the resulting subtrees were found to be highly variable, with some subtrees terminating after just two or three further rounds of branching while others extended over 20–30 rounds (Figure 1c).

Combining the results of EdU incorporation, as a marker of proliferation, and whole‐mount imaging of the whole mammary gland, the relative abundance of “active” terminal end‐buds was found to steadily diminish during puberty (Scheele et al., 2017), suggesting that terminal end‐buds progressively and collectively exit cell cycle during this phase. But what underpins such network heterogeneity? Does the complexity arise from the early specification of mammary stem cells with variable proliferative potential, or do mechanical, chemical or other environmental cues influence distinct fate decisions of equipotent mammary stem cell pools? To discriminate between these possibilities, evidence was sought for changes in the potency of terminal end‐buds during pubertal growth. However, notably, after the initial specification of the rudimentary ductal tree, over the remaining course of pubertal development, the average length and width of ductal segments remained approximately constant as a function of branching index – the latter defined as the minimum number of branches between a given ductal segment and the origin of the ductal tree. Moreover, the proliferative activity of terminal end‐buds that remain in cycle, as assayed by the constituent fraction of EdU+ cells in the end‐bud, also remained approximately constant over the developmental time course (Scheele et al., 2017). Together, these results suggested that the potency and proliferative activity of cycling mammary stem cells remains largely unchanged during the phase of pubertal growth. So, if terminal end‐buds, and their constituent stem and progenitor cells, remain equipotent during puberty, what is the source of ductal network heterogeneity?

During puberty, the localization of cell proliferation to terminal end‐bud regions allied with the network topology suggests that the choice between terminal end‐bud bifurcation (in which the number of active mammary stem cells is doubled) and “termination” (in which mammary stem cells and their immediate progeny collectively exit cell cycle) is not predetermined in an intrinsic, deterministic fashion, but is made stochastically. To test this conjecture, probabilities can be assigned to these events as a function of branch index, i.e. at a given generation, with probability *q*, a terminal end‐bud becomes inactive (with all cells exited from cell cycle) while, with probability *1‐q*, an active terminal end‐bud undergoes a symmetrical bifurcation, replicating the size, potency and activity of cells in the newly formed end‐buds (Figure 1d). Then, empirically, from the statistical ensemble of mammary gland networks, the variation of the average probability *q* with branch index was determined. In itself, such an assignment does not challenge a “model” of stochastic growth. To determine whether such a dependence provides the statistical basis of the network organization, one must further check that networks generated from this statistical rule faithfully predict the statistical organization of the reconstructed glands and their constituent subtrees.

Implementing this program, it was found that the termination probability rose monotonically from zero at the lowest branch levels becoming saturated at around *q* ≈ 1/2 for the later phase of pubertal development (Figure 1d). In other words, over much of the developmental time window, active terminal end‐buds appear to evolve according to a simple statistical rule in which, with approximately equal probability, they duplicate through ductal bifurcation, or terminate through collective cell cycle exit. But can this dependence alone predict the complexity of the mammary ductal network? Notably, within this framework, an estimate of the frequency of terminal end‐buds that remain in cycle agrees well with that measured experimentally based on short‐term EdU incorporation (Scheele et al., 2017). Moreover, using this empirical statistical rule to estimate the subtree persistence – the chance that subtrees survive to a given branch index – and the subtree size distribution, the stochastic model provided a good quantitative prediction of the experimental measurements (Scheele et al., 2017).

Together, these observations suggested that the complex network topology of the mouse mammary gland epithelium is defined by statistical rules that operate at the ductal scale. But what is happening at the cellular scale to affect the collective dynamics of actively proliferating terminal end‐buds? What is the size and sublineage potential of the constituent mammary stem cell pool? And how is the near‐balance between terminal end‐bud bifurcation and termination controlled?

## 3 UNBIASED CLONAL LINEAGE TRACING REVEALS THE MULTIPLICITY AND POTENCY OF MAMMARY STEM CELLS DURING PUBERTY

To address the cellular basis of mouse mammary gland development during puberty, it makes sense to deploy a genetic lineage tracing strategy to trace the long‐term fate behavior of individual cells (Desai, Brownfield, & Krasnow, 2014; Van Keymeulen et al., 2011). To trace the fate of cells in an unbiased manner, emphasis was placed on a multicolor cell labelling strategy based on the confetti reporter system under the control of a ubiquitous Rosa26 promoter – the *R26‐CreERT2;R26‐Confetti* model (Scheele et al., 2017). In this approach, the transient expression of Cre recombinase, activated by injection of tamoxifen, leads to the excision of a stop cassette resulting in the random hereditary expression of one of four fluorescent reporter genes (cytoplasmic RFP, YFP, CFP and nuclear GFP) in individual basal and luminal cells.

To interpret the results of the clonal assay, the singular nature of ductal growth in the mouse mammary gland is beneficial: With clonal labelling induced during puberty, cells in the rudimentary tree that have already exited cell cycle will appear as a “speckled” pattern of randomly labelled single‐cell clones of variable color. By contrast, proliferative cells in the terminal end‐buds that are labelled on induction divide and differentiate, giving rise to marked progeny that are laid down in the trailing ducts, providing a “historical” record of fate decisions made by cells at the ductal tip during development. Indeed, this highlights the need to combine lineage tracing at cellular resolution with whole‐organ reconstructions, as clones induced at the terminal end‐buds are expected to become dispersed through the entire mammary gland. Then, by resolving the sublineage identity and the statistical distribution of clonal imprints on the walls of the ducts and at the terminal end‐bud, insight can be gained into the multiplicity, sublineage potential and fate behavior of proliferative cells at the terminal end‐bud. To trace the dynamics of cells during the phase of ductal branching morphogenesis, labelling was induced in animals at 3 weeks of age and tissue fixed at 8 weeks, the end of puberty, when the expansion of the ductal network is complete.

Following low‐frequency (yet still mosaic) induction of tissue, whole‐mount reconstruction of the mammary gland epithelium revealed three kinds of clonal pattern (Scheele et al., 2017). Close to the origin of the ductal tree, as expected, a speckled pattern of individual labelled basal and luminal cells was visible (Figure 1e). In regions adjacent to the rudimentary tree, formed soon after induction, a mosaic of confetti labelled cell clusters were found within individual ducts, indicative of contributions made by clonally labelled mammary stem and progenitor cells marked within the same terminal end‐bud at the time of induction. These are the clonal footprints left behind as cells undergo the last rounds of cell duplication as they leave the end‐bud region before finally exiting cell cycle. Finally, in regions more remote from the rudimentary tree, the random segregation of confetti colors leads to progressive coarsening of the clonal distribution, with a gradual transition towards monoclonal labeling of ducts, where just one color or less becomes visible in distal ductal subtrees (Figure 1e). This behavior reflects a phase of clonal segregation and enrichment, characterized by “neutral” clonal dynamics. Before considering the quantitative information encoded within the clone size dependences, further qualitative features follow.

First, detailed analysis of mammary gland epithelium in whole‐mount stained for the basal or luminal cell markers, Keratin 14 and Keratin 8, respectively, allowed clones to be resolved by size and cell composition. From clonal maps showing both the representation of confetti labelled cells and their cell identity, it was apparent that, in line with similar clonal tracing studies (Davis et al., 2016; Lloyd‐Lewis, Davis, Harris, Hitchcock, & Watson, 2018; Van Keymeulen et al., 2011; Wuidart et al., 2016), by this stage of development, mammary stem cells have already become “compartmentalized”, lineage‐restricted to the basal or luminal compartments. However, based on these observations alone, further sublineage restriction within these compartments could not be ruled out. Indeed, recent clonal tracing studies targeting embryonic development show that the earliest phase of mammary growth (E12‐E18) is characterized by progressive sublineage restriction, with initially bipotent cells becoming gradually restricted to the basal (Wuidart et al., 2018) and luminal sublineages (Lilja et al., 2018), with luminal cells then becoming sublineage‐restricted into ER+ and ER‐ compartments (Rodilla et al., 2015). Whether there is further sub‐compartmentalization remains an open question, calling for lineage tracing studies based on targeted assays. Equally, given the capacity of basal cells to reacquire bipotency in response to damage or injury, the potential that a tiny minority bipotent population that survives during pubertal development but escapes clonal labelling can never be completely ruled out (Rios, Fu, Lindeman, & Visvader, 2014). However, taken together, evidence from the wide variety of clonal tracing studies suggests that mouse mammary gland pubertal development relies predominantly on the activity of sublineage‐restricted cells.

But what fraction of proliferative cells in terminal end‐buds function as self‐renewing mammary stem cells? To address this question, measurement of the relative fraction of clonally labelled cells in ductal subtrees provided the means to estimate the effective stem cell number in terminal end‐buds: In particular, if a given active terminal end‐bud plays host to a total of *N* equipotent stem cells, basal or luminal, each will contribute on average a fraction of *1/N* cells to the resulting “daughter” subtree. Notably, when averaged across multiple mice, estimates of this fraction for the basal and luminal compartments were found to be only marginally less than the total number of basal and luminal cells (estimated at 93 and 172, respectively) in each terminal end‐bud (Scheele et al., 2017). This suggests that, during puberty, the vast majority of proliferative cells at the terminal end‐bud belong to the “self‐renewing” stem cell pool.

Further analysis of the size distributions of basal and luminal sub‐clones at different branch generations indicated convergence towards a simple, exponential, dependence. By its nature, an exponential distribution of clone sizes is characterized by just one parameter, the average clone size. From this behavior, it therefore follows that sublineage‐restricted mammary stem cells must constitute populations that, in the medium term, function as equipotent pools (Scheele et al., 2017). However, given that monoclonal conversion occurs very slowly throughout development, this finding does not rule out the potential for further sub‐compartmentalization of the basal and luminal stem cell populations (Lilja et al., 2018; Wuidart et al., 2018), nor heterogeneity in their short‐term proliferative potential. Finally, the ratio of basal and luminal stem cells was found to be proportional to the fraction of basal and luminal cells in ducts, indicating that their respective clonal outputs are also similar.

However, measurement of the average size of sub‐clones in ducts as a function of the position of similarly‐labelled cells in proximate active terminal end‐buds shows that only clones with cells at the border of the end‐bud contribute to the immediate expansion of the adjacent duct, while clones with marked cells only at the tip make little or no contribution on average (Scheele et al., 2017). This finding suggests that, during rounds of ductal bifurcation, mammary stem cells move reversibly between states “primed” for renewal at the tip and biased for cell cycle exit at the border of the terminal end‐bud, echoing the organization of the intestinal crypt (Ritsma et al., 2014). Consistently, intravital imaging of the terminal end‐buds of the pubertal mammary gland revealed extensive cell rearrangements (Scheele et al., 2017), which would allow short‐term positional priming and molecular heterogeneity to be resolved into long‐term functional equipotency. Such behavior suggests that terminal end‐buds may constitute a niche‐like environment that maintains the self‐renewal potential of cells at the tip. Once cells move out of the niche, they are driven out of cycle. Subsequent lineage tracing results showing that specific cell populations, such as Blimp1 +  cells (Elias, Morgan, Bikoff, & Robertson, 2017), become enriched during pubertal growth would be consistent with markers enriching for terminal end‐bud‐located cells at the time of induction; although more work will be needed to better characterize molecularly and functionally terminal end‐bud cell subpopulations.

In summary, based on the statistics of the ductal network and the clonal tracing data, during pubertal development, it follows that the majority of cycling cells in terminal end‐buds function as self‐renewing sublineage‐restricted basal or luminal mammary stem cells, giving rise to a steady output of short‐lived progenitors with limited proliferative capacity, that fuel ductal elongation. Then, through a near‐balanced process of effectively stochastic terminal end‐bud bifurcation and termination, mammary stem cells act collectively to specify the complex irregular epithelial network. Folding during bifurcation allows stem cells to switch reversibly between border and tip regions of the end‐buds, reassigning fate bias, leading to long‐term equipotency of heterogeneous stem cell pools. But what regulates the near‐balance of terminal end‐bud bifurcation and termination during puberty? How could such stochastic termination events be regulated?

## 4 MAMMARY GLAND DUCTAL MORPHOGENESIS AS A BRANCHING‐ANNIHILATING RANDOM WALK

**Figure 2:**
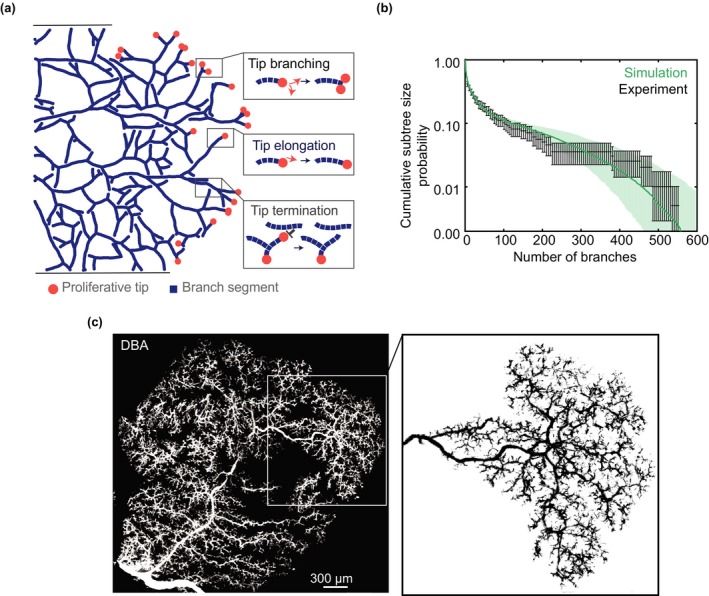
Unifying model of branching morphogenesis. (a) Schematic illustrating the branching‐annihilating random walk model. In this model, ductal morphogenesis involves a self‐organizing principle based on three local rules: (i) Ductal proliferation at tips drives a process of ductal elongation; (ii) ducts can bifurcate stochastically, leading to duplication of active tips; and (iii) active tips terminate when they encounter maturing ducts. (b) Comparison of the cumulative subtree size distribution obtained from the statistical analysis of mouse mammary glands (points) with that predicted by the model (lines) as depicted in (a). (c) Ductal network of mouse pancreas revealed by staining tissue with Dolichos biflorus agglutinin (DBA) at E18.5. Panels (a,b) are adapted from Figures presented in Hannezo et al., 2017; while panel (c) is adapted from Sznurkowska et al., 2018

To understand the mechanisms that regulate the collective cell dynamics of terminal end‐buds, the large‐scale spatial organization of the ducts was found to be revealing. Branching morphogenesis of mouse mammary gland takes place in a largely two‐dimensional setting, where the frequency of ductal crossovers is low (Hannezo et al., 2017). This observation led to the conjecture that, during pubertal development, terminal end‐buds may grow and branch at a constant rate, but terminate when exposed to secreted factors released from maturing ducts (Figure 2a) – a dynamic that, in the language of non‐equilibrium statistical physics, constitutes a “branching‐annihilating random walk”. Since the trajectories of tips are taken to follow a persistent random walk, this provides a framework in which termination events are effectively stochastic at the network level, although they are tightly regulated at the single tip level. To this process, a further condition was imposed that required end‐buds to terminate at the boundary of the fat pad. While the general topology of the resulting network mirrored qualitatively the observed structures (Figure 2a), the value of the model lay in its ability to predict quantitatively the statistical organization of the ductal network.

Remarkably, this model, which depends only on one (measurable) key parameter, the ratio of the branching to elongation rate, predicted the statistics of the branched ductal network, from the evolution of branch probability, *q*, with ductal index, to the distribution of subtree size and persistence (Figures 1d and 2b and Hannezo et al., 2017). Indeed, the stochastic character of the branching probability, a simplifying assumption within the framework of the model, was consistent with the measured distribution of branch lengths, which were seen to fall onto a strikingly exponential distribution (Hannezo et al., 2017). Further analytical insight can be gleaned from the development of a coarse‐grained or hydrodynamic theory of network growth. By defining the density of active terminal end‐buds and inactive immobile ducts, the branching dynamics takes the form of a reaction‐diffusion system (Hannezo et al., 2017). In this framework, it becomes apparent that, during the growth phase, the system becomes self‐organized into a soliton‐like front – a “Fisher‐KPP pulse” – of active terminal end‐buds on the periphery of the growing network that travels in a directional manner at constant speed, leaving in their wake a constant density of inactive ducts. Given this self‐organizing behavior, the observed convergence towards balanced fate of ductal growth becomes easy to understand: With active terminal end‐buds localized at the boundary, for each ductal bifurcation, on average, one daughter branch pioneers virgin territory while the other collides with the trailing network and terminates. Notably, as well as these qualitative dependences, from measurements of the growing ductal network, this minimal model could predict quantitatively the constant density of maturing ducts and pulse of active tips, including the “universal” ratio of the exponential decay constants between the advancing and trailing edge of the active pulse (Hannezo et al., 2017).

But what about statistical measures relating to fluctuations of the network? As a driven non‐equilibrium system, statistical fluctuations of ductal density are expected to be large. In the parlance of statistical physics, these are known as “giant density fluctuations” and take the form of a power‐law dependence of density fluctuations on the average ductal density with an exponent larger than one‐half, the value expected for a purely random distribution. Indeed, comparison of the data and the model revealed a strikingly power‐law dependence with the same elevated exponent of approximately two‐thirds.

Altogether, these findings suggested that the growth of the mammary gland ductal network is consistent with a minimal self‐organizing principle based on local rules. By correlating cell cycle exit with exposure to local factors released from maturing ducts (including TGFβ), the sequential process of ductal branching and elongation leads to the specification of a ductal network of constant average density. Could such a model based on the branching‐annihilating random walk be a general paradigm of ductal morphogenesis?

## 5 DUCTAL MORPHOGENESIS OF THE MOUSE KIDNEY AND PANCREAS

In mouse, the kidney develops as an outgrowth of the nephric duct that invades into the mesenchyme through a process of dichotomous ductal branching. Then, in a complex and cooperative process, the mesenchyme differentiates into epithelial tubes (nephron segments) that connect tips to form the basic filtration units of the kidney. In vitro studies involving the plating in two‐dimensions of two embryonic kidney explants next to each other revealed an arrest of tip growth between tips and ducts, preventing collisions and ductal crossovers (Davies, Hohenstein, Chang, & Berry, 2014). The overall structure and dynamics of such two‐dimensional kidney explants was thus accurately predicted by the same branching‐annihilating random walk theory used for mammary glands (Hannezo et al., 2017). Interestingly, tip arrest in these explants was shown to be dependent on Bmp7, a member of the TGFβ super‐family (Davies et al., 2014), which hints that the core findings from mammary gland could be translatable in other organs. Therefore, based on published data on the detailed structure of three‐dimensional kidney morphogenesis in vivo (Sampogna, Schneider, & Al‐Awqati, 2015), the question of whether there is a statistical basis to the branching network topology was addressed (Hannezo et al., 2017). Notably, in the three‐dimensional system, as well as the measured ratio of ductal elongation to branching rates, a second parameter had to be considered – the contact distance within which active tips become terminated against maturing ducts.

Once again, comparison of the ductal network statistics obtained from measurements made during embryonic development with numerical simulations generated from the branching‐annihilating random walk paradigm showed remarkably good agreement over a range of developmental time points (Hannezo et al., 2017). In particular, the data indicated that, although branching was seemingly stereotypic early in development, with most tips belonging to similar generations, this changed markedly post E15.5 (Sampogna et al., 2015), with considerable widening of the tip generation numbers, consistent with the predictions of the model. Quantitatively, by adjusting the two parameters of the theory – the ratio of the branching and elongation rates, and the contact radius of tip annihilation, good quantitative agreement was found for the variation of termination probability with branch index, as well as the distribution of branch number, subtree size and subtree persistence across a range of developmental time points. Indeed, these results suggest that the early phase of symmetric branching may not be a distinct phase of growth, as traditionally thought, but may be a natural consequence of the three‐dimensional branching dynamics where the chance of meeting a neighbor early in development is diminished.

Interestingly, a concomitant analysis of the branching rules of kidney morphogenesis up to E15.5 (Lefevre et al., 2017) has proposed an alternative hypothesis. In this framework, it has been argued that ductal organization results from asymmetrical branching rules where, upon each branching event, one tip would branch again soon after, while the other, potentially because of its close contact to neighboring tips/ducts, would be delayed in its branching capacity. How such an intrinsic, contact‐induced, delay process would result in stereotypically delaying one tip but not the other upon branching remains unclear. Unfortunately, since both models predict very few “tip terminations” pre‐E15 (around 10% in the case of the branching‐annihilating random walk), it is difficult to discriminate between these two competitive hypotheses using pre‐E15 data alone. Frustratingly, around E15, when differences in model predictions would begin to impact, the two different experimental datasets yield different levels of branching heterogeneity, a discrepancy that will need to be resolved. However, branching data from later time points (E15.5–E19.5, Sampogna et al., 2015) reveals a dramatic widening of the subtree size distributions and tip generation numbers, which can be well‐fit by the branching‐annihilating random walk paradigm (Hannezo et al., 2017). This contrasts with a model based on deterministic tip delay, which would predict that all tips should have comparable generation numbers (within a small variance of three to four generations).

An outstanding question would thus be to understand the molecular basis for such late‐stage tip generation heterogeneity. Although nephrogenesis had previously been proposed to suppress branching (Costantini & Kopan 2010; Sweeney, Lindström, & Davies, 2008), and could have been a candidate to track terminations (Hannezo et al., 2017), results from a recent study suggest that the genesis of nephrons does not correlate with delays in branching (Short et al., 2018). This argues that density‐dependent feedback on branching/elongation would occur via other mechanisms, leaving open the question of the cellular and molecular nature of tip termination and/or delay in vivo. Analysis of larger data sets, time windows and mouse models will be required to resolve the basis of kidney branching morphogenesis.

In a parallel study, the basis of ductal morphogenesis of mouse pancreas was addressed using a similar approach to the mouse mammary gland (Sznurkowska et al., 2018). In adult, the mouse pancreas forms a quasi‐two‐dimensional structure in which acinar cells lie anchored to the tips of an intricate and complex ductal network, which is interspersed with islets of Langerhans (Shih, Wang, & Sander, 2013). Mouse pancreas development initiates around E8.5 and evolves through a complex two‐stage process involving an early phase of plexus formation followed by an extended phase of plexus remodeling, which has been proposed to result from optimization of fluid‐transport rules (Dahl‐Jensen et al., 2018), and tip‐driven branching morphogenesis from the pancreas periphery (Bankaitis, Bechard, & Wright, 2015 and Figure 2c). To study the cellular basis of pancreas development, a clonal cell lineage tracing assay was combined with morphometric measurements of the large‐scale ductal organization.

Notably, measurements of the ductal subtree sizes revealed a distribution that, when rescaled by the average, overlapped closely with that obtained from studies of the mouse mammary gland (Sznurkowska et al., 2018). These findings suggest the basis of ductal morphogenesis in pancreas may involve the same branching‐annihilating random walk paradigm. This conclusion was reinforced by unbiased lineage tracing using the *R26‐CreERT2;R26‐Confetti* reporter system, which revealed qualitatively the existence of “tree‐shaped” acinar and ductal clones (i.e. clones closely tracking a single subtree in a monoclonal manner), and quantitatively a close correspondence between the statistical distribution clone and subtree sizes. This is expected from a branching process driven by self‐renewing “stem‐like” cells localized at or near the tips of active ducts. Although this analysis alone doesn't determine the exact transition point between central plexus remodeling and peripheral branching morphogenesis, a strength of the branching and annihilating framework is that it is self‐organized, and thus insensitive to initial conditions such as the starting geometry of the remodeled plexus (Sznurkowska et al., 2018).

At the cellular level, pancreatic precursors become increasingly sublineage‐restricted during embryonic development, with tripotent cells giving rise to self‐renewing bipotent ductal‐islet precursors and lineage‐restricted acinar precursors which co‐localize at the growing ductal tips, duplicating through serial rounds of ductal branching. However, in contrast to mammary gland, inspection of clonal imprints on the maturing postnatal day (P)14 tissue suggests that the abundance of self‐renewing cells is almost two orders of magnitude smaller than that found in the mammary gland. With confetti labelled ducts showing drift to monoclonality over just a few rounds of ductal branching, the size of self‐renewing ductal population was estimated to be just a handful of cells with similar estimates for tip‐localized self‐renewing acinar precursors.

## 6 DISCUSSION AND PERSPECTIVES

In summary, these studies suggest that, at the organ scale, the morphogenesis of a variety of ductal epithelia is rooted in a mechanism based on a simple local self‐organizing principle, based on the branching‐annihilating random walk. At the cellular scale, the cooperative dynamics of sublineage‐restricted self‐renewing precursors – “stem‐like” cells – drive a stochastic process of ductal bifurcation and elongation. These findings suggest a niche‐based pattern of regulation, similar to that found in the adult intestinal crypt (Lopez‐Garcia, Klein, Simons, & Winton, 2010), in which local factors at the ductal tip support the renewal potential of cells.

The apparent ubiquity of the branching mechanism begs the question whether other strategies are possible and/or deployed, in other tissue types or even at the single‐cell level in the case of branched neurons (Fujishima, Horie, Mochizuki, & Kengaku, 2012). Within the framework of the branching‐annihilating random walk, ducts fill space approximately uniformly in a self‐organized manner dependent on local rules that remain invariant during the branching process. This invariance of the regulatory program – a major evolutionary benefit – comes at the expense at imperfections or inefficiencies of the resulting branched structure; as mentioned above, the branching‐annihilating random walk process leaves behind chance voids leading to giant density fluctuations, or even worse, can lead to the catastrophic and premature extinction of the entire growing tree. In the context of the ductal tissues targeted in this review, such small imperfections may be of no consequence: In the mammary gland, genesis of alveoli from the ductal cell walls during pregnancy can efficiently expand and fill the interstitial regions between the ducts; in the kidney and pancreas, extensive proliferation and remodeling during the secondary phase of development can reorganize the positions of ducts into a more efficient space‐filling pattern. However, in other tissue types, such heterogeneities in the ductal network organization may present challenges, while developmental disorders may be mapped on more catastrophic extinction events.

In an alternative strategy, a much more efficient pattern can be generated by adjusting the branch length continuously with branch index leading to a more regular fractal‐like geometry with a high packing density. Studies of the ductal organization of mouse lung show evidence of a stereotypic pattern of early branching (Metzger, Klein, Martin, & Krasnow, 2008) based on a similar kind of organization, with geometric structures regulated by side‐branching patterns leading to power‐law scaling of ductal sizes with branch index (Horsfield, 1990). Whether these stereotypic patterns and rules are conserved down to the finest branch scales, or whether the later phases of branching are governed by a simpler statistical paradigm remains in question.

A striking and conserved feature of the mammary gland and pancreas development is the apparent cooperativity of sublineage‐restricted self‐renewing cells. In the former, basal‐ and luminal‐restricted mammary stem cells act cooperatively to generate the ductal epithelium, becoming proportionately expanded through ductal bifurcation. Similarly, in the pancreas, self‐renewing ductal‐islet‐restricted precursors act in concert with self‐renewing acinar‐committed precursors at the ductal tips, duplicating through serial rounds of branching. How is this cooperativity enforced? One possibility is that, in common with the mouse adult trachea or developing lung, basal and luminal cells (viz. club cells in the trachea) act as a niche for each other, allowing the relative size of the compartments to achieve a stable equilibrium (Nabhan, Brownfield, Harbury, Krasnow, & Desai, 2018; Pardo‐Saganta et al., 2015).

However, the problem of how sublineage‐restricted stem cells control their ratio questions the identity of other niche factors that promote self‐renewal activity. One possibility is that stromal cells surrounding the ductal tips – fibroblasts and endothelial cells – secrete factors that inhibit cell cycle exit (or differentiation). However, to ensure the long‐term persistence of renewing cells, such factors would have to co‐move with the elongating tip and be duplicated or recruited during ductal bifurcation – in essence, “pulling” the terminal end‐bud or tip like the proverbial “carrot tied to the donkey”. Alternatively, the maturing ducts themselves may secrete a factor that drives cells to exit cycle (or differentiate) – “pushing” the terminal end‐bud forward. Evidence in favor of key secreted factors from both the local tip environment (FGFs, etc.) as well as the maturing ducts (TGFβ, etc.) are present, suggesting that both mechanisms may act in concert.

Finally, to what extent does the branching‐annihilating random walk model provide true mechanistic insight? The answer to this question is likely to be subjective. The branching‐annihilating random walk model provides a *predictive* understanding of branching dynamics and so, for many must be considered as *mechanistic*. Moreover, by surrendering information at the cellular and molecular scale, it affords a unifying or “universal” description of the large‐scale dynamics. Put differently, multiple viable mechanisms of molecular or cellular regulation could lead to the same dynamics at the ductal scale, belonging to the class of branching‐annihilating random walks. However, for others, without a detailed understanding of the transcription factors and signaling molecules that effect the regulation of cell fate decisions, the model falls short – possibly well‐short – of real mechanistic understanding. In the end, this debate is difficult to reconcile.

In general, mechanistic understanding of a given phenomenon must be tailored to the appropriate level of abstraction – physicists speak of “phenomenology”. For example, we may understand the nature and action of a morphogenic program without resolving its microscopic basis. And such understanding may provide the means to frame targeted questions into the more microscopic level of description. In this sense, the problem of branching morphogenesis may serve as an exemplar for how to study and define the basis of emergent or collective behaviors in cell biological systems.
